# Do Genetic Polymorphisms Affect Fetal Hemoglobin (HbF) Levels in Patients With Sickle Cell Anemia Treated With Hydroxyurea? A Systematic Review and Pathway Analysis

**DOI:** 10.3389/fphar.2021.779497

**Published:** 2022-01-21

**Authors:** Rahyssa Rodrigues Sales, Bárbara Lisboa Nogueira, Jéssica Abdo Gonçalves Tosatti, Karina Braga Gomes, Marcelo Rizzatti Luizon

**Affiliations:** ^1^ Graduate Program in Genetics, Institute of Biological Sciences, Federal University of Minas Gerais, Belo Horizonte, Brazil; ^2^ Department of Clinical and Toxicological Analyzes, Faculty of Pharmacy, Federal University of Minas Gerais, Belo Horizonte, Brazil; ^3^ Department of Genetics, Ecology and Evolution, Institute of Biological Sciences, Federal University of Minas Gerais, Belo Horizonte, Brazil

**Keywords:** *BCL11A* gene, fetal hemoglobin (HbF), genetic polymorphisms, hydroxyurea (HU) therapy, pathway analysis, pharmacogenetics, sickle cell anemia (SCA), systematic review

## Abstract

Hydroxyurea has long been used for the treatment of sickle cell anemia (SCA), and its clinical effectiveness is related to the induction of fetal hemoglobin (HbF), a major modifier of SCA phenotypes. However, there is substantial variability in response to hydroxyurea among patients with SCA. While some patients show an increase in HbF levels and an ameliorated clinical condition under low doses of hydroxyurea, other patients present a poor effect or even develop toxicity. However, the effects of genetic polymorphisms on increasing HbF levels in response to hydroxyurea in patients with SCA (Hb SS) have been less explored. Therefore, we performed a systematic review to assess whether single-nucleotide polymorphisms (SNPs) affect HbF levels in patients with SCA treated with hydroxyurea. Moreover, we performed pathway analysis using the set of genes with SNPs found to be associated with changes in HbF levels in response to hydroxyurea among the included studies. The systematic literature search was conducted on Medline/PubMed, EMBASE, Cochrane Central Register of Controlled Trials, Cumulative Index to Nursing and Allied Health Literature (CINAHL), Scopus, and Web of Science. Seven cohort studies were included following our inclusion and exclusion criteria. From the 728 genetic polymorphisms examined in the included studies, 50 different SNPs of 17 genes were found to be associated with HbF changes in patients with SCA treated with hydroxyurea, which are known to affect baseline HbF but are not restricted to them. Enrichment analysis of this gene set revealed reactome pathways with the lowest adjusted *p*-values and highest combined scores related to VEGF ligand–receptor interactions (R-HSA-194313; R-HSA-195399) and the urea cycle (R-HSA-70635). Pharmacogenetic studies of response to hydroxyurea therapy in patients with SCA are still scarce and markedly heterogeneous regarding candidate genes and SNPs examined for association with HbF changes and outcomes, suggesting that further studies are needed. The reviewed findings highlighted that similar to baseline HbF, changes in HbF levels upon hydroxyurea therapy are likely to be regulated by multiple loci. There is evidence that SNPs in intron 2 of *BCL11A* affect HbF changes in response to hydroxyurea therapy, a potential application that might improve the clinical management of SCA.

**Systematic Review Registration: **(https://www.crd.york.ac.uk/prospero/display_record.php?RecordID=208790).

## Introduction

Sickle cell anemia (SCA) is a global health problem, and approximately 300,000 infants are born with SCA every year (Azar and Wong, 2017). SCA is defined as a monogenic hemoglobin disorder caused by homozygosis for A-to-T transversion at codon 7 (c.20A > T, p.E7V) in the hemoglobin subunit beta (*HBB*) gene (den Dunnen and Antonarakis, 2000; Steinberg, 2008). The pathophysiology of SCA is directly related to polymerization of deoxygenated hemoglobin (HbS; α2βS2), leading to a cascade of pathologic events including erythrocyte sickling, vaso-occlusion, and hemolysis (Kato et al., 2018). It is important to note that higher levels of fetal hemoglobin (HbF; α2γ2) ameliorate clinical outcomes and hematological parameters of SCA, since it reduces HbS concentration and inhibit copolymerization between hemoglobin tetramers (Kato et al., 2018). Notably, higher persistent HbF concentration is often observed in patients with SCA than in subjects without SCA (Lettre and Bauer, 2016).

Hydroxyurea (HU) was approved by the U.S. Food and Drug Administration for the treatment of adults with severe SCA in 1998, and it has been associated with improved survival for both adults and children with SCA, as reviewed elsewhere (McGann and Ware, 2015). The clinical effectiveness of HU is related to the induction of the production of HbF, but it is not restricted to it. HU selectively kills cells in the bone marrow and increases the number of erythroblasts producing HbF, which inhibits the intracellular polymerization of HbS and prevents the sickling process in erythrocytes, thereby decreasing the number of sickled cells (McGann and Ware, 2015). Erythrocytes with high HbF have longer survival, thereby attenuating hemolysis (Steinberg, 1999). Furthermore, HU increases the hemoglobin levels; reduces neutrophils, monocytes, and reticulocytes; and alters the expression of adhesion molecules in the endothelium and the generation of nitric oxide. These hematological changes decrease the risk of vaso-occlusion in patients with SCA (Steinberg, 1999; McGann and Ware, 2015; Rigano et al., 2018).

Because HU has dose-related effects, the laboratory and clinical benefits of HU were shown to be optimized when dimensioned for the maximum tolerated dose (MTD). Almost all patients with SCA show a significant increase in HbF concentration at the MTD (McGann and Ware, 2015). The American Society of Hematology guideline panel suggests HU therapy with at least 20 mg/kg/day at a fixed dose or the MTD (DeBaun et al., 2020). However, there is substantial interpatient variability both in the MTD itself and in the percentage of HbF (% HbF) achieved (Ware et al., 2011; McGann and Ware, 2015). For example, the % HbF achieved with the MTD of HU reaches 10–15% in some patients, but it can reach 40% in other patients (Ware et al., 2011). Moreover, while some patients tolerate high HU doses, such as 30–35 mg/kg/day, others develop severe myelosuppression even at lower doses (Lettre et al., 2008). These findings suggest that important individual differences on pharmacokinetics and pharmacodynamics, and genetic factors contribute to the phenotypic variability in both the dosing and response to HU therapy (McGann and Ware, 2015). However, the effect of genetic polymorphisms on increasing HbF levels in response to HU therapy in patients with SCA has been less explored.

Therefore, the aim of the present study was to perform a systematic review to assess whether genetic polymorphisms affect HbF levels in patients with SCA treated with HU. In addition, we performed pathway analysis using the set of genes which had single-nucleotide polymorphisms (SNPs) that were found to be associated with changes in HbF levels in response to HU therapy among the studies included in the systematic review.

## Materials and Methods

This study was conducted according to the Cochrane Handbook for Systematic Reviews of Interventions (Higgins et al., 2020), and the results were reported in accordance with the Meta-analysis Of Observational Studies in Epidemiology (MOOSE) checklist (Stroup et al., 2000). The protocol of the current study was registered on the International Prospective Register of Systematic Reviews [PROSPERO (CRD42020208790); https://www.crd.york.ac.uk/prospero/display_record.php?RecordID=208790].

### Search Strategy

The search strategy was defined based on the PECO question: Participants (P) = Sickle cell anemia patients (HbSS); Exposition (E) = Minor alleles; Control (C) = Major alleles of genetic polymorphisms and; Outcomes (O) = Fetal hemoglobin levels. A literature review was conducted by searching the electronic databases EMBASE, Medline/PubMed (Medical Literature Analysis and Retrieve System Online), Cochrane Central Register of Controlled Trials (CENTRAL), Cumulative Index to Nursing and Allied Health Literature (CINAHL), Scopus, and Web of Science (WoS) to identify studies published until July 2021. The initial search included the Medical Subject Headings (MeSH) entry terms: “Anemia, Sickle Cell”; and “Hydroxyurea”; and “Polymorphism, Genetic” or “Amplified Fragment Length Polymorphism Analysis” or “Polymorphism, Single Nucleotide,” or “Polymorphism, Restriction Fragment Length”; and “Fetal Hemoglobin,” which were then included for a high-sensitivity search strategy in Medline/PubMed (Supplementary Table S1).

The same terms were used to search for gray literature and conference proceedings. The reference lists of included articles were also checked to identify additional relevant citations. All potentially eligible studies were considered for review, regardless of the language and publication date.

### Inclusion and Exclusion Criteria

The inclusion criteria were restricted to studies that described the pharmacogenetics of response to HU therapy in patients with SCA measured by HbF levels (primary outcome). We included only cohort studies that examined patients with the SS genotype, with a minimum age of three y at the time of HU initiation and with a minimum period of six months of HU therapy.

We excluded studies that did not differentiate patients with SCA from patients with another sickle cell disease (SCD), studies that focused on haplotypes and not on individualized SNPs, and studies that did not examine whether SNPs affect HbF levels in patients with SCA treated with HU. Review articles, conference proceedings, case reports, and commentary studies were also excluded.

### Study Selection and Data Extraction

Initially, the studies retrieved from the databases were input into a single electronic library, and duplicates were excluded using EndNote^®^ software. Two reviewers (R.R.S. and B.L.N.) independently analyzed the titles and abstracts of the articles retrieved, reviewed the full text of the published articles, and used a standard data extraction protocol. Any disagreements between the two reviewers were resolved by a third reviewer (J.A.G.T.).

The extracted data from selected studies included study design, country, sample size, follow-up duration, median/mean age of participants, gender of patients, eligibility criteria, median/mean of HU dose, changes in HbF levels after HU therapy, genes, and polymorphisms associated with the primary outcome. The associated genes found in the included studies were used for pathway analysis.

### Assessment of Bias Across Studies

The quality assessment of included studies was carried out independently by two reviewers (R.R.S. and B.L.N.), following the approach of the Joanna Briggs Institute for the synthesis of evidence (Moola et al., 2020), and any disparity between the two reviewers was resolved by a third reviewer (J.A.G.T.). The approach indicates the application of critical assessment tools used in systematic reviews, in which the checklist for cohort studies is applied (Moola et al., 2020). The instrument is structured around eleven questions, in which the selected studies were evaluated: 1) the two groups were similar and recruited from the same population; 2) how they were similarly measured to designate exposed and unexposed groups as people; 3) exposure was measured in a valid and reliable manner; 4) confounding factors have been identified; 5) the instrument was created to deal with confounding factors; 6) the groups were free of the outcome at the beginning of the study; 7) the results were measured in a valid and reliable way; 8) the follow-up time was reported and long enough for the results to occur; 9) the follow-up was complete and, if not, whether the reasons for the loss of follow-up were obtained and explored; (10) the instrument was used to deal with incomplete follow-up; and 11) statistical statistics was applied. The answer options for signaling questions are 1) yes, 2) no, 3) unclear, and 4) not applicable (Moola et al., 2020).

### Gene Set Enrichment Analysis and Pathway Analysis

After the data extraction, we manually curated the genes which had SNPs that were found to be associated with changes in HbF levels in patients with SCA treated with HU among the seven studies included in the systematic review (listed in Table 2). Next, we interrogated this gene set for significant well-curated signaling pathways obtained from the Reactome Pathway Knowledgebase (Jassal et al., 2020). The pathways found were sorted both by the adjusted *p*-values ranking <0.05, which were calculated using a Benjamini–Hochberg method (Benjamini and Hochberg, 1995), and the combined scores according to the gene set enrichment analysis web server Enrichr (Kuleshov et al., 2016; Xie et al., 2021).

## Results

### Study Selection

**FIGURE 1:**
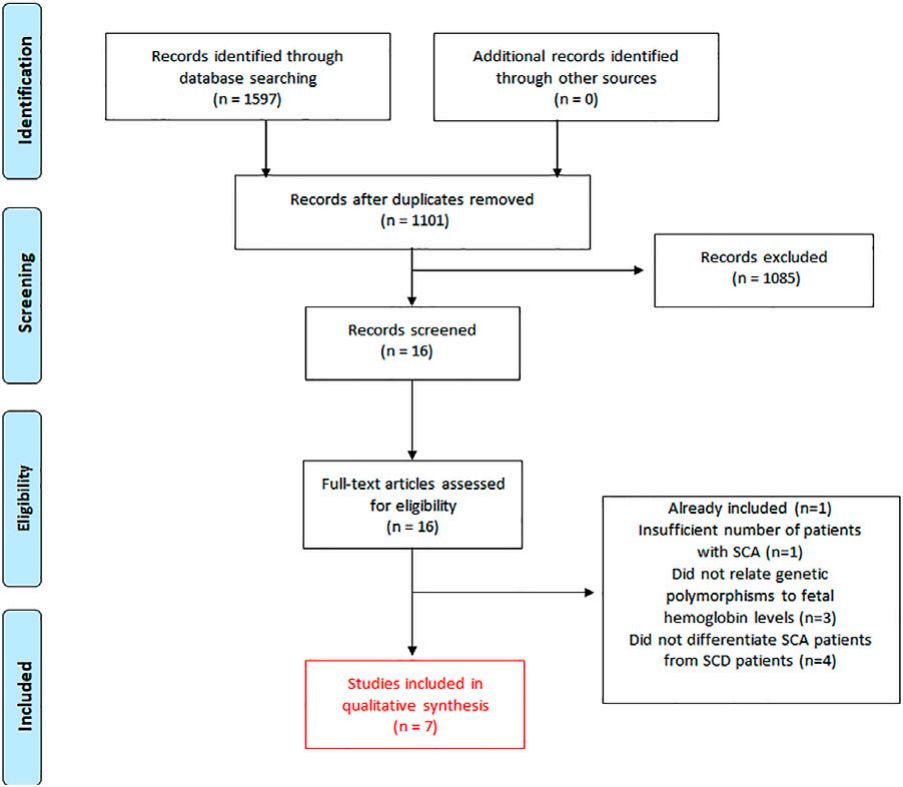
Flow diagram of the literature search and the study selection for this systematic review of published articles related to the effect of genetic polymorphisms on HbF levels in patients with SCA (Hb SS) treated with hydroxyurea.

We identified 1,597 records in the initial search (Figure 1). After the exclusion of duplicates, 1,101 articles were selected for title and abstract analyses. Of these, 1,085 articles were subsequently excluded due to the following reasons (as stated before in the “exclusion criteria”): 1) studies that focused on haplotypes rather than individualized SNPs; 2) studies that did not differentiate patients with SCA from patients with another SCD; or 3) studies that did not focus on SNPs related to HbF levels in patients with SCA treated with HU. Subsequently, 16 full-text articles were thoroughly assessed for inclusion. Following review, nine articles were removed due to the following reasons (Figure 1): One cohort study had an insufficient number of patients with SCA (Sclafani et al., 2016). Three studies did not assess whether the SNPs affected HbF levels (Italia et al., 2010; Zhu et al., 2017; Yahouedehou et al., 2020). Four studies did not differentiate patients with SCA from patients with SCD (Borg et al., 2012; Gravia et al., 2016; Chondrou et al., 2017; Elalfy et al., 2017). One study was part of an oral session and their results were later published in an original article already included in this systematic review (Wyszynski et al., 2004). Finally, seven cohort studies were included in this systematic review (Ma et al., 2007; Kumkhaek et al., 2008; Ware et al., 2011; Green et al., 2013; Sheehan et al., 2014; Friedrisch et al., 2016; Aleluia et al., 2017) (Figure 1).

### Characteristics of the Included Studies

Out of the seven included studies, five studies had data from the United States and two studies had data from Brazil. Sample size in the included studies ranged from 42 to 174 patients with SCA. The publication date ranged from 2007 to 2018, and the sample mean age ranged from 8.1 to 21 y. The mean dose of HU ranged from 19 to 27.1 ± 4.3 mg/kg/day. The mean duration of treatment with HU ranged from 13.4 to 102 months (Table 1). Two studies calculated the change in HbF levels for each patient from baseline to the MTD (delta HbF) (Ware et al., 2011; Friedrisch et al., 2016), while other four studies used the increment in HbF after treatment with HU (final HbF) (Ma et al., 2007; Kumkhaek et al., 2008; Green et al., 2013; Sheehan et al., 2014), and one study calculated from the baseline to maximum HbF during treatment with HU (Aleluia et al., 2017).

**TABLE 1:** Characteristics of the seven cohort studies included in the systematic review, which examined the effects of genetic polymorphisms on fetal hemoglobin (HbF) levels in patients with SCA treated with hydroxyurea (HU).

Author, data; country	Sample (n)	Average age (years)*	Gender (M/F)	Dose of HU (mean ± SD; mg/kg/day)	Time of follow-up on HU therapy (months)	HbF changes	HbF measurement	Number of genes (SNPs) studied	Multiple test correction
Friedrisch et al. (2016); Brazil	111	21 ± 14 (from 4 to 54)	38/62	23 ± 7.6	Minimum of 6	∆ MTD HbF (%)^a^	Capillary electrophoresis	3 (6)	Not applied
Ware et al. (2011); United States	88	9.6 ± 4.8	57/31	23.9 ± 5.1	Minimum of 6	∆ MTD HbF (%)^b^	HPLC	Not informed (331)	Applied
Aleluia et al. (2017); Brazil	42	15.2 ± 11.1	70/71	15 (47.6%)	Mean of 13.4 ± 9.7	Not informed	HPLC	3 (6)	Not applied
				20 (23.8)					
				25 (26.2%)^**^					
Green et al. (2013); United States	38	12.5 ± 4.9	57/60	25.3 ± 3.0	Minimum of 6	∆ HbF (%)^c^	HPLC	9 (20)	Applied
Kumkhaek et al. (2008); United States	32	Not informed	Not informed	Not informed	Minimum of 8	∆ HbF (% and g/dl)^b^	HPLC	1 (20)	Not applied
Sheehan et al. (2014); United States	Discovery cohort (171)	10.4 ± 4.5	Not informed	25.1 ± 4.5	Minimum of 6	∆ MTD HbF (%)^b^; Final HbF	HPLC	Whole exome	Unclear
	Validation cohort (130)	8.1 ± 4.0	Not informed	27.1 ± 4.3	Minimum of 6	∆ MTD HbF (%)^b^; Final HbF	HPLC	24 (25)	Unclear
Ma et al. (2007); United States	137	Not informed	Not informed	Not informed	Minimum of 21	∆ HbF (% and g/dl)^a^	Alkali denaturation	29 (320)	Not applied

Abbreviations: HbF, fetal hemoglobin; M, male; F, female. All selected studies were part of cohort studies.

* age at the time of hydroxyurea initiation.

** dose (case percentage).

a (∆HbF = MTD HbF—baseline HbF).

b (∆HbF = final HbF—baseline HbF).

c (∆HbF = maximum HbF—baseline HbF).

Overall, 728 genetic polymorphisms were assessed for their association with changes in HbF levels in patients with SCA under treatment with HU, and 11 candidate genes were the most examined in the seven included studies. Four studies examined *BCL11A* and the *HBSIL-MYB* intergenic region (Ware et al., 2011; Green et al., 2013; Sheehan et al., 2014; Friedrisch et al., 2016; Aleluia et al., 2017). Three studies focused on arginase 1 and 2 (*ARG1* and *ARG2*) genes (Ma et al., 2007; Ware et al., 2011; Green et al., 2013). Two studies evaluated the secretion-associated Ras-related GTPase 1A (*SAR1A*) gene (Ma et al., 2007; Green et al., 2013). Two studies examined the *XmnI* gene (Ware et al., 2011; Friedrisch et al., 2016). The Fms-related receptor tyrosine kinase 1 (*FLT1*), hydroxyacid oxidase 2 (*HA O 2*), nitric oxide synthase 1 (*NOS1*), and olfactory receptor family 51 subfamily B member 5 and 6 (*OR51B5/6*), genes were mentioned only once by two studies (Ma et al., 2007; Aleluia et al., 2017).

Regarding the quality assessment according to the Joanna Briggs Institute checklist (Supplementary Table S2), one of the seven articles answered affirmative in all the 11 questions. Five studies responded affirmative to ten out of the 11 questions. One study answered affirmative to six out of the 11 questions, while three of the questions were negative and two were not applicable.

### Pharmacogenetics of Response to HU Therapy in Patients With SCA

Among the included studies, a cohort study involving 137 adult African-Americans with SCA from the Multicenter Study of Hydroxyurea in Patients With Sickle Cell Anemia (MSH) examined the association of 320 tagging SNPs from 29 candidate genes with changes in HbF concentrations (measured by %, g/dL and F-cell count) after two years of HU treatment (Ma et al., 2007). Candidate genes were involved in the regulation of DNA transcription, cell proliferation/differentiation, and drug metabolism functions. The daily dose of HU started at 15 mg/kg and increased by 5 mg/kg each week up to the MTD, which means 35 mg/kg, unless toxicity was established. The authors found 17 SNPs to be associated with % HbF change and 20 SNPs to be associated with change of absolute HbF (g/dl), most of them being located in introns or untranslated genomic regions. The SNPs found to be associated with the higher mean of squared were rs2182008 in the *FLT1* gene and rs10483801 in the *ARG2* gene, which is involved in the metabolism of HU. This MSH cohort study performed analysis with age, sex, and *β-globin* haplotypes as covariates and showed several SNPs in other genes as predictors for HbF response. In the random forest algorithm, the SNP rs21822998 in *FLT1* and the SNP rs9376173 in the phosphodiesterase 7B (*PDE7B*) gene had a higher mean of squared residuals as predictors for % HbF and absolute HbF, respectively (Ma et al., 2007).

The hypothesis for a later pharmacogenetic study (Kumkhaek et al., 2008) was supported by an experimental study on the molecular mechanisms underlying the increase in HbF levels induced by HU (Tang et al., 2005). The authors searched for differential gene expression in human adult erythroid cells and identified a small guanosine triphosphate (GTP)–binding protein, whose secretion is associated with Ras-related (SAR) protein, as a specific gene induced by HU. SAR was shown to play a key role in *γ*-globin (*HBG*) gene induction by promoting cell apoptosis and G1/S-phase arrest by the reduction of PI3K and extracellular signal–regulated kinase phosphorylation and increasing p21 and GATA-binding protein 2 expression (Tang et al., 2005). From these experimental findings, variations of the *SAR1A* gene were hypothesized to explain differences in individual responses to HU treatment (Kumkhaek et al., 2008). The authors tested whether 20 variants in the upstream promoter region, exon 1, and intron 1 of *SAR1A* were associated with HbF changes in response to HU compared to baseline in 32 adults with SCA from the Sickle Cell Pulmonary Hypertension Screening Study, prospectively followed up during two y of HU therapy. The SNP rs231099 was found to be associated with the change in % HbF, and the SNPs rs2310991, rs76901216, rs76901216, and rs4282891 were found to be associated with the change in absolute HbF (g/dl). The intronic SNP rs4282891 showed stronger association, which is phylogenetically conserved in vertebrates (Kumkhaek et al., 2008).

The Hydroxyurea Study of Long-Term Effects (HUSTLE) was a prospective clinical trial for pediatric patients with SCA receiving HU designed to understand the interpatient variability in the responses and toxicities to HU (Ware et al., 2011). A candidate gene study was conducted to carry out pharmacogenetic analyses for the HU end points of % HbF and the MTD. The dose administrated in patients who were included before beginning HU therapy (new cohort; *n* = 88) started with 15 mg/kg/day, and it was escalated every eight weeks to a maximum dose of 30 mg/kg/day or the MTD, with careful monitoring of blood counts every two weeks. If hematologic toxicity occurred twice at the same dose, the MTD was set at 2.5 mg/kg below the toxic dose. Pharmacogenetic analyses included 331 SNPs in candidate genes that were selected based on their presumed pharmacogenetic and pharmacodynamic effects of HU. The *ARG1* rs175999586 and *ARG2* rs2295644 SNPs were associated with the change in % HbF between baseline and MTD. The SNP rs1427407 of the *BCL11A* gene was associated with the MTD, but none was associated with the MTD after adjustment for baseline % HbF (Ware et al., 2011).

The association of several SNPs with HbF levels induced by HU was also examined in a multi-site observational study of 117 pediatric patients (5–21 y), which was mainly composed of SCA patients (93% of HbSS and 7% of Sβ0-thalassemia) (Green et al., 2013). SNPs of *BCL11A*, *HBSIL-MYB*, *HBB*, hemoglobin subunit beta (*HBE*), *OR51B6*, glucagon-like peptide 2 receptor (*GLP2R*), *SAR1A*, *ARG1*, and *ARG2* genes, which were reported as associated with baseline HbF levels, were also examined for their association with HbF under HU therapy (“maximum HbF” and “delta HbF,” from baseline to maximum). Clinical indications for HU therapy were comparable across sites (nearly all for repetitive painful crises and/or acute chest episodes) at least for six months. Stable dosing was reached at three months or near maximal dose by absolute neutrophil count criteria, excluding data from subjects on less than 20 mg/kg/day, even for dose-limiting toxicity. The SNPs of *BCL11A* (rs766432, rs11886868, rs4671393, and rs7557939), *HBE* (rs7130110), and *GLP2R* (rs12103880) were associated with maximum HbF under HU. Only the SNP rs7130110 of *HBE* was associated with delta (∆) HbF (Green et al., 2013).

A cohort composed of 171 patients from the HUSTLE study and 51 patients from the Stroke with Transfusions Changing to Hydroxyurea (SWiTCH) (called “discovery”) was examined to identify genetic predictors of HbF response to HU, with focus on protein coding regions (Sheehan et al., 2014). Whole-exome sequencing was performed in two prospective pediatric cohorts with robust HbF phenotype data and a standardized dose escalation regimen to the MTD, which were genotyped for SNPs of *BCL11A* (rs1427407, rs4671393, rs11886868, and rs7599488) and *HBSIL-MYB* (rs9399137 and rs9402686). HbF responses to HU were measured by maximum % HbF at the MTD (“final HbF”) or the change in % HbF from baseline to final (“delta HbF”). The patients had baseline HbF measured after three y of age. The HU therapy initiated with 20 mg/kg, and then dose was escalated to mild myelosuppression. The average age of the patients at the time of HU initiation was 10.4 ± 4.5 y. However, they found no associations of *BCL11A* or *HBSIL-MYB* variants with HbF response. Whole-exome sequencing identified 13 and 12 variants associated with final HbF and delta HbF (*p*-value < 5 × 10^−4^), respectively. Although these associations did not achieve the genome-wide significance level (*p*-value < 1.3 × 10^−6^), they did provide suggestive signals (Sheehan et al., 2014). By using functional prediction methods, the authors identified that 13 variants associated with HbF response to HU were predicted to introduce an amino acid change, inducing damage in the protein structure or function (Sheehan et al., 2014). These 13 variants were then genotyped in a validated cohort composed of 130 unrelated children with SCA receiving HU at Texas Children’s Hospital Hematology Center for at least six months prior to the MTD. One variant (P840R; rs61743453) in the spalt-like transcription factor 2 (*SALL2*) gene was associated with higher delta HbF and with final HbF in the discovery and the validated cohorts, respectively. A meta-analysis combining the discovery and validation cohorts (*n* = 301) found that the P840R variant was associated with both delta HbF (*p* = 8.30 × 10^−4^) and final HbF (*p* = 1.48 × 10^−4^) (Sheehan et al., 2014).

A cohort of 121 patients with SCA under regular HU therapy for at least six months at the Sickle Cell Center of the Clinical Hospital from Porto Alegre (Southern Brazil) was examined for the effect of genetic variants at the major loci modifier of baseline HbF on HU-induced HbF levels (Friedrisch et al., 2016). Patients who received any other drugs stimulating HbF (e.g., erythropoietin) or blood transfusion within three months prior to the study were excluded. HbF measurements were obtained before HU (baseline HbF) and at the MTD (MTD HbF), and the change from baseline to the MTD (delta HbF) was calculated for each patient. The association tests were performed by linear regression analyses adjusted for age at start HU, gender, and absolute neutrophil count at MTD. SNPs of hemoglobin subunit gamma 2 (*HBG2*) (rs7482144), *BCL11A* (rs1427407, rs4671393, and rs11886868), and *HBS1L-MYB* (rs9399137 and rs9402686) were assessed, and patients with variations in SNPs of *BCL11A* had a favorable probability of producing more HbFs in response to HU treatment (Friedrisch et al., 2016).

A cross-sectional study of 141 patients with SCA, including 99 patients under HU treatment, followed up at the Sickle cell Disease Reference Center in Itabuna (Northeastern Brazil) was examined for the role of *HBB* haplotypes and SNPs at quantitative trait loci (QTL) associated with baseline HbF in regulating HbF response to HU (Aleluia et al., 2017). HbF measures were not performed in patients with clinical manifestations of vaso-occlusive crisis or transfusions in the last three months. Patients were genotyped for *β* ^*S*^ *-globin* gene cluster haplotypes and SNPs of *BCL11A* (rs6732518 and rs766432), *HBS1L-MYB* (rs11759553 and rs3595442), and *OR51B5/6* (rs4910755 and rs7483122). Almost 48% of the patients received 15 mg/kg/day, while 23.8% received 20 mg/kg/day and 26.2% received 25 mg/kg/day. The only patient who received the maximum dose of 35 mg/kg/day was excluded from the analysis. Multiple linear regression analysis adjusted for gender and age were used to investigate the association of SNPs with HbF induction, and the authors concluded that homozygous subjects for the minor allele of rs766432 of *BCL11A* had higher increases in HbF (Aleluia et al., 2017).

**FIGURE 2:**
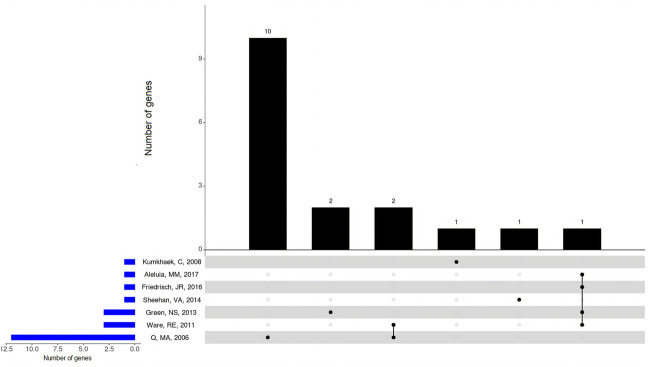
Overlapping genes with SNPs associated with changes on HbF levels in the included studies involving patients with SCA (Hb SS) under HU therapy. *BCL11A*, *ARG1*, and *ARG2* genes showed overlap between included studies. Upset plot showing the total number of genes with SNPs found to be associated with changes on HbF levels identified in each study (horizontal bars), and the number of genes exclusive to one study or shared by different studies (vertical bars). Black dots below vertical bars indicate genes quantified.

In summary, seven studies involving patients with SCA treated with HU identified 50 SNPs of 17 different genes to be associated with HbF changes from baseline to HU (Table 2; Figure 2). Five out of the seven included studies examined SNPs of *BCL11A*, of which four (80%) found SNPs to be associated with HbF changes (Ware et al., 2011; Green et al., 2013; Friedrisch et al., 2016; Aleluia et al., 2017). These studies confirmed the associations of the *BCL11A* SNPs rs1427407 (Ware et al., 2011; Friedrisch et al., 2016), rs4671393, rs11886868 (Green et al., 2013; Friedrisch et al., 2016), rs766432 (Green et al., 2013; Aleluia et al., 2017), and rs7557939 (Green et al., 2013). In addition, two studies found associations for SNPs of *ARG1* (rs17599586, rs2781667, and rs1799586) and *ARG2* (rs2246012, rs2295644, rs10483801, and rs10483802) (Ma et al., 2007; Ware et al., 2011). Among them, only the SNP rs1799586 of *ARG1* was found to be associated with HbF changes in the two studies (Ma et al., 2007; Ware et al., 2011).

**TABLE 2:** Genes and chromosomes for the 50 different SNPs found to be associated with changes on HbF [described as delta (Δ) % HbF, Δ HbF (g/dl), maximum tolerated dose (MTD) % HbF, or maximum HbF] in response to hydroxyurea therapy in the seven cohort studies included in the systematic review. *The SNP rs17599586 of *ARG1* and three SNPs of *BCL11A* (rs1427407, rs4671393, and rs11886868) were found to be associated by two different cohort studies.

Gene	Chromosome	SNP	HbF response	Reference
*ARG1*	6	*rs17599586	Δ % HbF; Δ HbF (g/dL)	Ma et al. (2007)
*ARG1*	6	rs2781667	Δ % HbF; Δ HbF (g/dL)	Ma et al. (2007)
*ARG1*	6	*rs17599586	Δ % HbF	Ware et al. (2011)
*ARG2*	14	rs2246012	Δ HbF (g/dl)	Ma et al. (2007)
*ARG2*	14	rs2295644	Δ % HbF	Ware et al. (2011)
*ARG2*	14	rs10483801	Δ % HbF; Δ HbF (g/dl)	Ma et al. (2007)
*ARG2*	14	rs10483802	Δ % HbF; Δ HbF (g/dl)	Ma et al. (2007)
*ASS*	9	rs7860909	Δ % HbF; Δ HbF (g/dl)	Ma et al. (2007)
*ASS*	9	rs10793902	Δ % HbF	Ma et al. (2007)
*ASS*	9	rs10901080	Δ % HbF	Ma et al. (2007)
*ASS*	9	rs543048	Δ HbF (g/dl)	Ma et al. (2007)
*BCL11A*	2	rs766432	Δ % HbF	Aleluia et al. (2017)
*BCL11A*	2	*rs1427407	MTD % HbF; Δ % HbF	Friedrisch et al. (2016)
*BCL11A*	2	*rs4671393	MTD % HbF	Friedrisch et al. (2016)
*BCL11A*	2	*rs11886868	MTD % HbF; Δ % HbF	Friedrisch et al. (2016)
*BCL11A*	2	rs766432	Maximum HbF	Green et al. (2013)
*BCL11A*	2	*rs11886868	Maximum HbF	Green et al. (2013)
*BCL11A*	2	*rs4671393	Maximum HbF	Green et al. (2013)
*BCL11A*	2	rs7557939	Maximum HbF	Green et al. (2013)
*BCL11A*	2	*rs1427407	MTD, mg/kg	Ware et al. (2011)
*FIGF*	X	rs6632521	Δ % HbF	Ma et al. (2007)
*FLT1*	13	rs9319428	Δ % HbF; Δ HbF (g/dl)	Ma et al. (2007)
*FLT1*	13	rs2182008	Δ % HbF; Δ HbF (g/dl)	Ma et al. (2007)
*FLT1*	13	rs3751395	Δ HbF (g/dl)	Ma et al. (2007)
*FLT1*	13	rs8002446	Δ % HbF; Δ HbF (g/dl)	Ma et al. (2007)
*FLT1*	13	rs2387634	Δ HbF (g/dl)	Ma et al. (2007)
*GLP2R*	17	rs12103880	Maximum HbF	Green et al. (2013)
*HA O 2*	1	rs10494225	Δ % HbF	Ma et al. (2007)
*HBE*	11	rs7130110	Maximum HbF; Δ % HbF	Green et al. (2013)
*MAP3K5*	6	rs9376230	Δ % HbF	Ma et al. (2007)
*MAP3K5*	6	rs9483947	Δ % HbF	Ma et al. (2007)
*NOS1*	12	rs7309163	Δ HbF (g/dl)	Ma et al. (2007)
*NOS1*	12	rs816361	Δ % HbF	Ma et al. (2007)
*NOS1*	12	rs7977109	Δ % HbF; Δ HbF (g/dL)	Ma et al. (2007)
*NOS2A*	17	rs1137933	Δ % HbF	Ma et al. (2007)
*NOS2A*	17	rs944725	Δ % HbF	Ma et al. (2007)
*PDE7B*	6	rs2327669	Δ HbF (g/dl)	Ma et al. (2007)
*PDE7B*	6	rs11154849	Δ HbF (g/dl)	Ma et al. (2007)
*PDE7B*	6	rs9376173	Δ HbF (g/dl)	Ma et al. (2007)
*PDE7B*	6	rs1480642	Δ HbF (g/dl)	Ma et al. (2007)
*PDE7B*	6	rs487278	Δ HbF (g/dl)	Ma et al. (2007)
*PIR*	X	rs2071182	Δ HbF (g/dl)	Ma et al. (2007)
*SALL2*	14	rs61743453	Δ % HbF	Sheehan et al. (2014)
*SAR1*	10	rs2310991	Δ % HbF; Δ HbF (g/l)	Kumkhaek et al. (2008)
*SAR1*	10	rs76901216	Δ HbF (g/dl)	Kumkhaek et al. (2008)
*SAR1*	10	rs76901220	Δ HbF (g/dl)	Kumkhaek et al. (2008)
*SAR1*	10	rs4282891	Δ HbF (g/dl)	Kumkhaek et al. (2008)
*TOX*	8	rs2693430	Δ HbF (g/dl)	Ma et al. (2007)
*TOX*	8	rs826729	Δ % HbF	Ma et al. (2007)
*TOX*	8	rs765587	Δ % HbF; Δ HbF (g/dl)	Ma et al. (2007)
*TOX*	8	rs9693712	Δ % HbF; Δ HbF (g/dl)	Ma et al. (2007)
*TOX*	8	rs172652	Δ % HbF	Ma et al. (2007)
*TOX*	8	rs380620	Δ % HbF; Δ HbF (g/dl)	Ma et al. (2007)
*TOX*	8	rs12155519	Δ HbF (g/dl)	Ma et al. (2007)

### Gene Set Enrichment Analysis and Pathway Analysis

**FIGURE 3:**
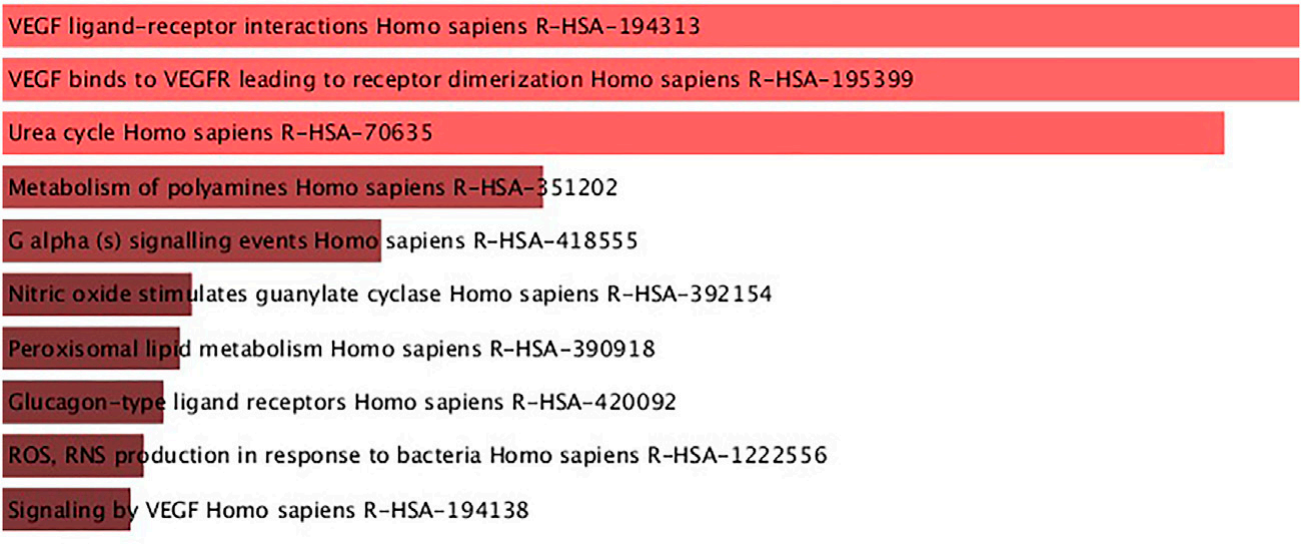
Reactome pathways obtained from enrichment analysis of the set of genes with SNPs found to be associated with HbF changes in patients with SCA treated with hydroxyurea. Pathways are ordered from top to bottom according to the lowest adjusted *p*-values, and light red bars indicate adjusted *p*-values < 0.001.

Reactome pathways were obtained from the enrichment analysis using the set of genes that had SNPs found to be associated with changes on HbF levels in patients with SCA under HU therapy (Figure 3; Supplementary Table S3). The reactome pathways with both lowest adjusted *p*-values and highest combined scores were related to VEGF binding, namely, “VEGF ligand–receptor interactions” (R-HSA-194313; adjusted *p*-value = 0.0002847; combined score = 4,826.43) and “VEGF binds to VEGFR leading to receptor dimerization” (R-HSA-195399; adjusted *p*-value = 0.0002847; and combined score = 4,826.43). Moreover, we obtained the reactome pathway “urea cycle” (R-HSA-70635; adjusted *p*-value = 0.0003048; combined score = 3,461.84)] (Figure 3; Supplementary Table S3). The reactome pathway “nitric oxide stimulates guanylate cyclase” (R-HSA-392154; Figure 3) ranked fourth but with a lower combined score (200.68; *p*-value = 0.02105; Supplementary Table S3).

## Discussion

Genetic variability in response to HU therapy is scarcely explored, despite almost 50 y of HU use and 30 y of the treatment of patients with SCA (Ware et al., 2011). Notably, the literature regarding the effects of genetic polymorphisms on HbF levels in patients with SCA (Hb SS) treated with HU is remarkably scarce. In this systematic review, only seven studies met the inclusion criteria.

Importantly, patient-specific factors, including age, concomitant diseases, diet, and genetic factors, contribute to the interindividual variability in drug efficacy and risk of adverse reactions, and genetic polymorphisms explain around 20–30% of the interindividual variability in drug response (Lauschke et al., 2017). Indeed, the knowledge of how genetic variation contributes to variation in drug response has been expanded (Gong et al., 2021), and guidelines for the clinical implementation of pharmacogenetics have been published (Relling et al., 2020).

### Single-Nucleotide Polymorphisms and HbF Modulation

As expected, some genes previously associated with baseline HbF and known for acting in the HbF regulation pathway were found to be associated with HbF changes in response to HU therapy in the included studies. There is evidence that SNPs of intron 2 of *BCL11A* affect HbF changes in patients with SCA treated with HU. Five out of the seven included studies examined the role of the three main QTLs associated with baseline HbF levels: *BCL11A*, *XmnI*, and *HBS1L-MYB* intergenic region (Ware et al., 2011; Green et al., 2013; Sheehan et al., 2014; Friedrisch et al., 2016; Aleluia et al., 2017). Noteworthy, BCL11A is a negative regulator of HbF expression. Subjects with variation in any of the established SNPs of *BCL11A* are known to have decreased *BCL11A* expression, which results in increased HbF production (Lettre et al., 2008; Bauer et al., 2013). *HBS1L-MYB* genes are expressed in the erythroid precursor cells (Lettre et al., 2008; Bauer et al., 2013). *HBS1L* encodes a protein with apparent GTP-binding activity and is involved in a variety of cellular processes, while *MYB* encodes a transcription factor for erythroid differentiation in hematopoiesis (Thein et al., 2007). The *HBS1L-MYB* intergenic region is known to contain several common QTLs associated with HbF levels and a long-range erythroid enhancer that regulates *MYB* expression by chromatin looping (Stadhouders et al., 2014). Finally, the *XmnI* restriction site at -158 position of the *HBG* gene is associated with an increased expression of *γ*-globin and higher HbF production (Sripichai and Fucharoen, 2016). Together, they account for approximately 20–50% of the variation in HbF levels in patients with SCA and β-thalassemia, and even in healthy adults (Galarneau et al., 2010).

Four out of five studies that examined SNPs of *BCL11A* found associations with HbF response (Ware et al., 2011; Green et al., 2013; Sheehan et al., 2014; Friedrisch et al., 2016). These SNPs (rs1427407, rs4671393, rs11886868, rs766432, and rs7557939) are located in intron 2 of *BCLL1A*, which is a region marked by functional elements. These SNPs are located nearby several DNase hypersensitive sites, which indicate a genomic region of open chromatin. Noteworthy, the critical SNP rs1427407 (G > T) falls within a +62 DNase I hypersensitive site, an erythroid enhancer of *BCL11A*, and overlaps a peak of GATA1 and TAL1 transcription factor–binding sites. Notably, the minor T-allele for the SNP rs1427407 disrupts the G nucleotide of a consensus sequence [CTG (n9)] enriched for GATA1 and TAL1 transcription factors. GATA1- and TAL1-binding sites are more frequent in the G-allele than T-allele in the primary erythroblast samples (Bauer et al., 2013). In agreement with our findings regarding the effect of SNPs of *BCL11A* on HbF response, a recent functional *in vitro* study based on gene editing comparative analysis showed that *BCL11A* is the most clinically relevant approach focused on HbF resurgence (Lamsfus-Calle et al., 2020). This functional information supports the effect of *BCL11A* SNPs on baseline HbF, but the way it affects the response to HU remains to be elucidated. Biological network analysis integrating effects of HU on gene expression in erythroid precursors could highlight pathways involved in this process.

The *Xmnl* variant in the *HBBP1* gene was also examined. One study found the SNP rs7482144 to be associated only with an increase in baseline HbF (Ware et al., 2011) but not with HbF changes to HU, while other studies excluded this SNP because it had a very low allele frequency (0.45%, only one heterozygous subject) in a Brazilian cohort (Friedrisch et al., 2016).

The SNPs rs9399137 and rs9402686 are located in the *HBS1L-MYB* intergenic region but were not found to be associated with the increase in HbF in two included studies (Sheehan et al., 2014; Friedrisch et al., 2016). *SAR1A*, a gene belonging to the small GTPase superfamily that encodes a GTP-binding protein called SAR1A, has been reported to be associated with *HBG* expression. The SNP rs2310991 of *SAR1A* was associated with the change in absolute HbF concentration (Kumkhaek et al., 2008). Conversely, other studies found no association of rs2310991 with posttreatment HbF levels (Kumkhaek et al., 2008; Green et al., 2013).

Our findings may potentially guide the selection of candidate gene regulatory sequences within these genomic regions to be validated by *in vitro* functional assays in cells treated with HU, such as luciferase reporter assays. Further studies may examine whether the variation in these SNPs would affect the activity of the gene regulatory element, such as an enhancer or a silencer. Therefore, the present findings can contribute to guide further functional studies, which may advance the research focused on genomics of HbF changes in response to HU therapy.

### Signaling Pathways Underlying HbF Changes in Response to HU Therapy

Pathway analysis using genes with SNPs found to be associated with HbF changes in patients with SCA treated with HU in the included studies revealed pathways underlying HbF changes in response to HU. For example, we found enrichment of the reactome pathway “urea cycle” (R-HAS-70635; Figure 3), which is directly related to arginine (Friebe and Koesling, 2003). Indeed, cytosolic arginase 1 is a canonical enzyme of the urea cycle. Arginase 2 was described to play a role in the regulation of the urea cycle arginine metabolism and in downregulation of nitric oxide synthesis (Mori, 2007). Arginase isoforms encoded by *ARG1* and *ARG2* genes were also related to the increase in HbF levels induced by HU (Ware et al., 2011). The SNPs rs175999586 and rs2295644 of *ARG1* and *ARG2* were associated with the changes in HbF, respectively. Notably, rs2295644 has been implicated in kidney disease, so it could affect the renal clearance of HU and possibly the dose of the MTD (Ware et al., 2011). Another *ARG2* SNP (rs10483801) was also associated with the absolute HbF change (Ma et al., 2007).

We also found the enrichment of the reactome pathway “nitric oxide stimulates guanylate cyclase” (R-HSA-392154; Figure 3). Noteworthy, HU was suggested to act as a nitric oxide donor in patients with SCA (King, 2004). Nitric oxide is synthetized from l-arginine, stimulates vasodilatation of the endothelium and disaggregation of platelet aggregates, and inhibits platelet activation, an important modulator of SCA pathophysiology (Radomski et al., 1987). Moreover, HU was shown to modulate the nitric oxide signaling pathway in erythrocytes, rheology of erythrocytes, and oxidative stress through its effects on HbF and possibly on nitric oxide bioavailability (Nader et al., 2018).

A complex regulatory environment determines the HbF concentration in the blood, as well as chromosome remodeling, transcription factors, erythropoiesis modulation, gene regulatory elements linked to the *β*-globin gene cluster, and the kinetics of erythroid cell differentiation and differential red cell survival (Ma et al., 2007). Therefore, there is a large opportunity for the genetic modulation of HbF production. Consistent with this complex regulation apparatus, even with the restricted number of studies, our systematic review suggests that there is huge heterogeneity in genetic elements modulating the HbF levels in response to HU treatment. Unfortunately, some genetic associations with HbF response have not been reproduced by other studies, and further investigations are needed to conclude their use in predicting HbF response to HU.

Dosing and monitoring regimens of HU have yet to be determined (Ware, 2010). The best results from treatment with HU are found when the dose is escalated to the MTD, improving laboratory variables and reducing clinical complications. The dose escalation of HU is a labored process that requires risk monitoring of cytopenias, mainly neutropenia, and the clinical response to treatment with HU may take up to six months after reaching the MTD (NIH US, 2014). Therefore, severe patients with clinical recommendation for HU might have to experience a long exposure time until deducing that the treatment with HU is ineffective. Therefore, the prediction of HbF induction in response to treatment with HU by using SNPs in the intron 2 of *BCL11A* gene may have potential clinical applicability in the management of SCA.

The induction of HbF is a powerful mechanism of action of HU. However, since several other mechanisms of actions are known, further research is needed to conclude whether such SNPs are able to predict a subgroup of patients as “responders” to HU. Noteworthy, these SNPs were previously associated with increased baseline HbF levels, milder hematological parameters, and lower risk of clinical complications (Sales et al., 2020). Interestingly, not all these associations were dependent on HbF. Therefore, future studies should evaluate if the SNPs located in intron 2 of the *BCL11A* gene are also able to distinguish patients who show a reduced rate of clinical complications when treated with HU from those patients who do not show this reduction. This evidence is of huge importance to assess the cost-effectiveness of the use of pharmacogenetic tests for these SNPs in the SCA management.

Some studies do not meet the inclusion criteria of this review due to the different genotypes of the study subjects (Borg et al., 2012; Chondrou et al., 2017; Elalfy et al., 2017). They involve other *β*-type hemoglobinopathies, and known differences in their hematological parameter could bias the review. However, these studies highlight specific points regarding pathways related to the HbF regulation. Two studies suggested that *KFL1* expression and the SNP rs3191333 of *KFL1* play a role in the HbF regulation and are biomarkers of HU response in *β*-type hemoglobinopathies. It makes biological sense, since KLF1 regulates *BCL11A* expression and the *γ*- to *β*-globin gene switching (Zhou et al., 2010). Further studies can confirm their influence in HU therapy in patients with SCA (Hb SS).

Another study suggests that the vascular endothelial growth factor (*VEGFA*) gene is a biomarker in *β*-type hemoglobinopathies severity and efficacy of HU therapy (Chondrou et al., 2017). These findings are in agreement with a study included in this systematic review that found SNPs in the *FLT1* gene, encoding VEGF receptor 1, associated with HbF changes by HU therapy in Hb SS patients (Ma et al., 2007). Interestingly, we found enrichment of two reactome pathways related to VEGF ligand–receptor interactions (R-HSA-194313 and R-HSA-195399; Figure 3). The binding of VEGF ligands to VEGFR receptors in the cell membrane triggers intracellular signaling cascades, which results in proliferation, survival, migration, and increased permeability of vascular endothelial cells (Matsumoto and Mugishima, 2006). It is important to SCA pathophysiology, since endothelial dysfunction plays a key role in sickle cell vasculopathy, as reviewed elsewhere (Wood et al., 2008).

Our systematic review highlighted the role of SNPs on HbF induction upon HU therapy. However, it is important to note that this is one of the several mechanisms underlying response to HU. Indeed, a previous systematic review reported on the molecular mechanisms of HbF induction by HU in SCD (Pule et al., 2015). The reviewed findings pointed out three main pathways: epigenetic modifications, signaling pathways involving HU-mediated response, and posttranscriptional pathways, focusing on regulation by small non-coding RNAs (miRNAs). In this context, several miRNAs were identified as differentially expressed in patients with SCD under HU treatment, most of them being functionally related to genes known to regulate HbF, including *BCL11A* (Mnika et al., 2019). Notably, an experimental study showed that downregulation of *BCL11A*, *MYB*, and *KLF1* induces *γ-globin* expression by miRNA-mediated mechanisms, and miR-26b directly interacted with the 3′-untranslated region of *MYB* (Pule et al., 2016). Since miRNAs have been associated with a multitude of regulatory mechanisms, their functions may add to the complex mechanisms underlying response to HU.

**FIGURE 4:**
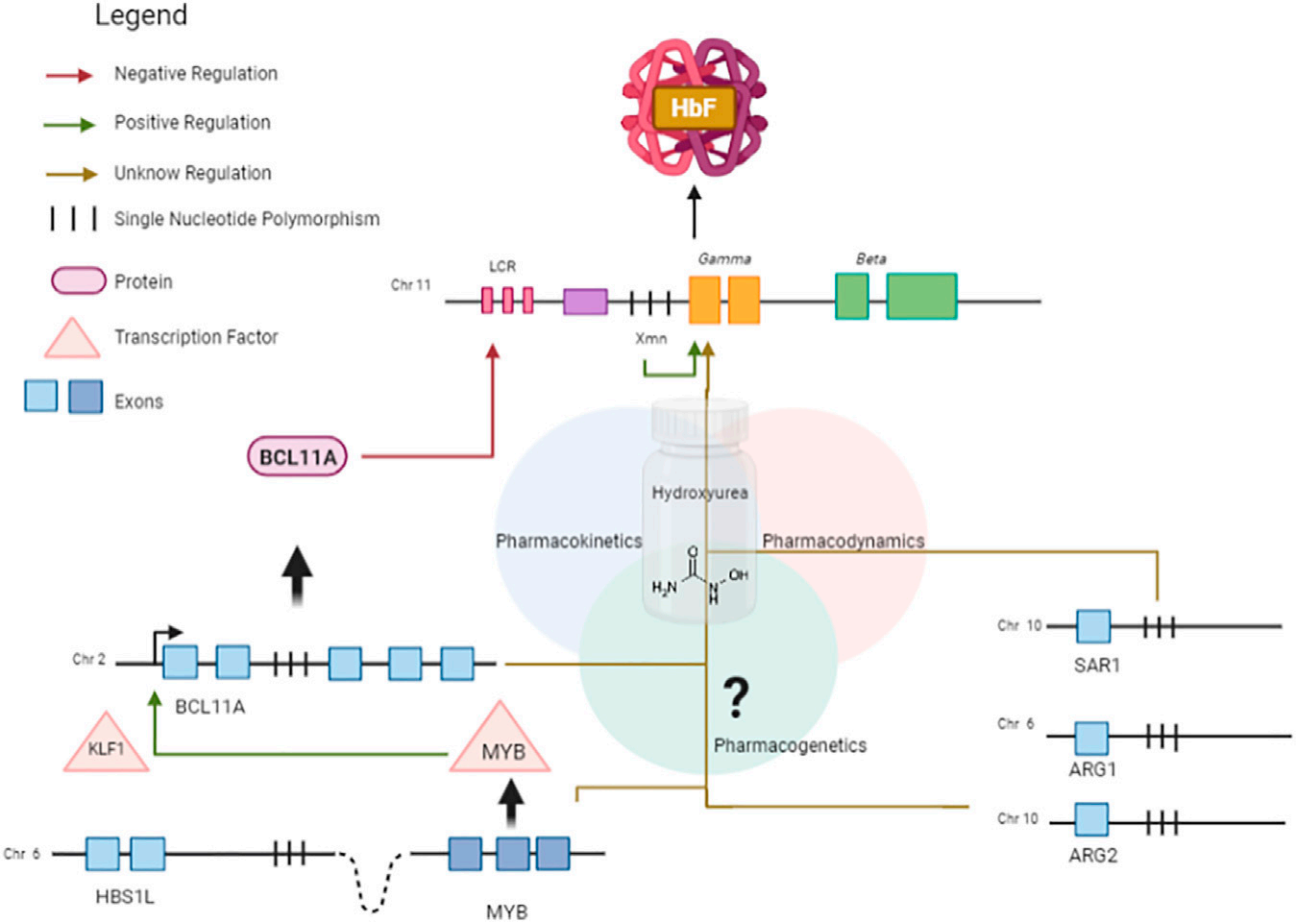
Schematic diagram showing HbF regulation in response to HU, an HbF-inducing drug. *BCL11A* is a master repressor of gamma genes. The erythroid transcription factor KLF1 activates the transcription of *BCL11A* by binding to its promoter region. The expression of *KLF1* is possibly regulated by MYB. The *HBS1L-MYB* intergenic region contains several trait-associated common genetic variations and a distal enhancer of *MYB*, promoting long-range transcription regulation by chromatin looping. *SAR1A* encodes a GTP-binding protein which has been reported to be associated with *γ*-globin expression. Variations in the candidate genes *ARG1* and *ARG2* are associated with HbF changes. Pharmacokinetic genes can affect absorption, distribution, and metabolism of HU.

In summary, the regulation of HbF involves both *cis*- and *trans*-regulatory elements, which interact in a complex network. HU promotes the induction of HbF, and the mechanisms by which it interacts with genetic modifiers of HbF affecting drug response are not fully understood. In this context, SNPs located within gene regulatory elements can have a major effect on differences in drug response (Luizon and Ahituv, 2015). A proposed schematic diagram to HbF regulation in response to HU is shown in Figure 4, including the functional findings of genes found in this systematic review as candidates to modify the HbF response to HU in patients with SCA.

### Confounding Factors

The first clinical manifestations of SCA appear along with the replacement of HbS by HbF (Rumaney et al., 2014). After 10 y, age is no longer an indicator of red cell deformability in patients with SCA; instead, this hemorheological parameter is mainly affected by the level of HbF, sex, and HU treatment (Thein et al., 2007).

The level of HbF is the best predictor of clinical severity of SCA (Wonkam et al., 2014). However, there is no threshold or value that characterizes the high and low baseline HbF levels for response to HU treatment. It was established that subjects who start with baseline HbF values between 5 and 10% can have a 2- to 3-fold HbF increase, whereas subjects with very low baseline HbF can have a 10-fold increase after treatment with HU (Wonkam et al., 2014). In the MSH cohort study, the baseline HbF was not predictive to HbF response to HU. On the other hand, baseline HbF was found as a predictor of the direction of association to % HbF at MTD (Ware et al., 2011). However, the change in HbF outcome in patients with SCA treated with HU was largely heterogeneous among the seven included studies, which examined different HbF outcomes in response to HU. Changes in HbF upon HU therapy was calculated as absolute HbF (g/dl), % HbF, and F-cell count from baseline until the MTD or a defined time of therapy (about 2 y). Although the % HbF and the amount of F cells are highly correlated, some patients with high levels of HbF develop severe complications of SCA probably due to the heterogeneous distribution of HbF among erythrocytes (Khandros et al., 2020). The number of F cells with polymer-inhibiting concentrations of HbF is likely to be a more accurate predictor of clinical benefits of HU therapy than HbF levels. However, the distribution of HbF among F cells is often unavailable, mainly in health centers of least developed countries. Using HbF under the MTD to calculate delta HbF probably provides the maximum level that the patient can achieve.

Patients with SCA experience several acute clinical events involving pronounced changes in hematological parameters (Novelli and Gladwin, 2016). Moreover, they commonly receive blood transfusion for treating and avoid a range of complications, which introduces biases on evaluating the association with hematological variables, including HbF. However, three of the seven included studies did not describe whether strategies were used to deal with these established confounding factors, which introduces bias in our analyses and constitute a limitation of this systematic review.

Although clinical experience of HU therapy for patients with SCA has been related for more than 25 y, there is still much questioning about the pharmacokinetics, pharmacodynamics, and pharmacogenetics of HU (Ware et al., 2011). To better understand the interpatient variability, polymorphisms in genes encoding drug-metabolizing enzymes and solute transporters were recently examined to learn their role in HU bioavailability and metabolism (Yahouedehou et al., 2020). The authors found evidence for the involvement of enzymes of the CYP450 family and catalases in the metabolism of HU, and the association between urea transporter-B (UTB) and response to HU in erythroid cells. SNPs in the *CYP2D6* (rs3892097), *CAT* (rs7943316 and rs1001179), and *SLC14A1* (rs2298720) genes were found to be linked to reduced metabolism or the elimination of HU, which may increase its therapeutic effects in patients with SCA (Yahouedehou et al., 2020). Unfortunately, this study did not examine the association between the SNPs with HbF response to HU, and thus, it was not included in the systematic review.

There was great heterogeneity in the patients’ age in the included studies. For example, one study examined patients aged 4–54 y (mean 21 ± 14 y) (Friedrisch et al., 2016). The average age of initiation of HU was 9.6 ± 4.8 y in another study (Ware et al., 2011).

The present study has layers of complexity linked to the multifactorial characteristic of the disease. The heterogeneity of HU dose, patient age, HbF outcomes in response to HU, and candidate genes brought limitations to the search and contributed to the result being only seven included studies. These findings highlight that the pharmacogenetics of response to HU in patients with SCA is a fertile field for further investigations.

## Conclusion

The literature about the pharmacogenetics of response to HU therapy in patients with SCA is highly heterogeneous regarding the chosen candidate genes and SNPs examined for the possible association with changes in HbF levels, and regarding the HbF outcomes measured during HU therapy. Nevertheless, the findings of the studies included in this systematic review point out two main conclusions. First, as well as the baseline HbF, changes in HbF levels in response to HU therapy are likely to be regulated by genetic variations on multiple loci. Second, there is evidence that SNPs located in intron 2 of the *BCL11A* gene affect HbF changes in patients with SCA treated with HU. However, further studies are needed to test whether such SNPs may also predict the success of the treatment in ameliorating other hematological parameters and reducing the incidence of clinical complications.

## Data Availability

The original contributions presented in the study are included in the article/Supplementary Materials; further inquiries can be directed to the corresponding authors.
