# Supplementary material for: Do Genetic Polymorphisms Affect Fetal Hemoglobin (HbF) Levels in Patients With Sickle Cell Anemia Treated With Hydroxyurea? A Systematic Review and Pathway Analysis
Source: Front Pharmacol. 2022 Jan 21;12:779497. doi: 10.3389/fphar.2021.779497 (PMC8814522; doi:10.3389/fphar.2021.779497)
Supplement: Supplementary file 1 [file DataSheet1.docx]

**Supplementary Table 1**. Complete search strategy, including the Medical Subject Headings (MeSH) terms and entry terms.

| PECO | MESH terms | Entry terms | Search Strategy |
| --- | --- | --- | --- |
| Population | Anemia, Sickle Cell | Anemias, Sickle Cell; Sickle Cell Anemias; Hemoglobin S Disease; Disease, Hemoglobin S; Hemoglobin S Diseases; Sickle Cell Anemia; Sickle Cell Disorders; Cell Disorder, Sickle; Cell Disorders, Sickle; Sickle Cell Disorder; Sickling Disorder Due to Hemoglobin S; HbS Disease; Sickle Cell Disease; Cell Disease, Sickle; Cell Diseases, Sickle; Sickle Cell Diseases | ("Anemia, Sickle Cell"[Mesh]) OR (Anemia, Sickle Cell[Text Word] OR Anemias, Sickle Cell[Text Word] OR Sickle Cell Anemias[Text Word] OR Hemoglobin S Disease[Text Word] OR Disease, Hemoglobin S[Text Word] OR Hemoglobin S Diseases[Text Word] OR Sickle Cell Anemia[Text Word] OR Sickle Cell Disorders[Text Word] OR Cell Disorder, Sickle[Text Word] OR Cell Disorders, Sickle[Text Word] OR Sickle Cell Disorder[Text Word] OR Sickling Disorder Due to Hemoglobin S[Text Word] OR HbS Disease[Text Word] OR Sickle Cell Disease[Text Word] OR Cell Disease, Sickle[Text Word] OR Cell Diseases, Sickle[Text Word] OR Sickle Cell Diseases[Text Word]) |
|  | Hydroxyurea | Hydroxycarbamid; Oncocarbide; Hydrea | ("Hydroxyurea"[Mesh]) OR (Hydroxyurea[Text Word] OR Hydroxycarbamid[Text Word] OR Oncocarbide[Text Word] OR Hydrea[Text Word]) |
| Exposition | Polymorphism, Genetic | Polymorphisms, Genetic; Genetic Polymorphisms; Genetic Polymorphism; Polymorphism (Genetics); Polymorphisms (Genetics) | ("Polymorphism, Genetic"[Mesh]) OR (Polymorphism, Genetic[Text Word] OR Polymorphisms, Genetic[Text Word] OR Genetic Polymorphisms[Text Word] OR Genetic Polymorphism[Text Word] OR Polymorphism (Genetics)[Text Word] OR Polymorphisms (Genetics)[Text Word]) |
|  | Amplified Fragment Length Polymorphism Analysis | AFLP Analysis; AFLP Analyses; Analyses, AFLP; Analysis, AFLP | ("Amplified Fragment Length Polymorphism Analysis"[Mesh]) OR (Amplified Fragment Length Polymorphism Analysis[Text Word] OR AFLP Analysis[Text Word] OR AFLP Analyses[Text Word] OR Analyses, AFLP[Text Word] OR Analysis, AFLP[Text Word]) |
|  | Polymorphism, Single Nucleotide | Nucleotide Polymorphism, Single; Nucleotide Polymorphisms, Single; Polymorphisms, Single Nucleotide; Single Nucleotide Polymorphisms; SNPs; Single Nucleotide Polymorphism | ("Polymorphism, Single Nucleotide"[Mesh]) OR (Polymorphism, Single Nucleotide[Text Word] OR Nucleotide Polymorphism, Single[Text Word] OR Nucleotide Polymorphisms, Single[Text Word] OR Polymorphisms, Single Nucleotide[Text Word] OR Single Nucleotide Polymorphisms[Text Word] OR SNPs[Text Word] OR Single Nucleotide Polymorphism[Text Word]) |
|  | Polymorphism, Restriction Fragment Length | RFLPs; Restriction Fragment Length Polymorphisms; Restriction Fragment Length Polymorphism; RFLP | ("Polymorphism, Restriction Fragment Length"[Mesh]) OR (Polymorphism, Restriction Fragment Length[Text Word] OR RFLPs[Text Word] OR Restriction Fragment Length Polymorphisms[Text Word] OR Restriction Fragment Length Polymorphism[Text Word] OR RFLP[Text Word]) |
| Outcome | Fetal Hemoglobin | Hemoglobin, Fetal; Hemoglobin F | ("Fetal Hemoglobin"[Mesh]) OR (Fetal Hemoglobin[Text Word] OR Hemoglobin, Fetal[Text Word] OR Hemoglobin F[Text Word]) |

**Supplementary Table 2**. Results of the quality assessment of the seven studies included in the systematic review according to the 11 questions of the Joanna Briggs Institute checklist.

| Citation | Q1 | Q2 | Q3 | Q4 | Q5 | Q6 | Q7 | Q8 | Q9 | Q10 | Q11 |
| --- | --- | --- | --- | --- | --- | --- | --- | --- | --- | --- | --- |
| Friedrisch *et al.*, 2016 | Yes | Yes | Yes | Yes | Yes | Yes | Yes | Yes | Yes | N/A | Yes |
| Ware *et al*., 2011 | Yes | Yes | Yes | Yes | Yes | Yes | Yes | Yes | Yes | N/A | Yes |
| Aleluia *et al*., 2017 | Yes | Yes | U | Yes | Yes | Yes | U | No | No | No | Yes |
| Green *et al.*, 2013 | Yes | Yes | Yes | Yes | Yes | Yes | Yes | Yes | Yes | N/A | Yes |
| Kumkhaek *et al.*, 2008 | Yes | Yes | Yes | Yes | Yes | Yes | Yes | Yes | Yes | Yes | Yes |
| Sheehan *et al.*, 2014 | Yes | Yes | Yes | Yes | Yes | Yes | Yes | Yes | Yes | N/A | Yes |
| Ma *et al.*, 2007 | Yes | Yes | Yes | Yes | Yes | Yes | Yes | Yes | Yes | N/A | Yes |

Abbreviations: U= Unclear N/A= Not applicable.

**Supplementary Table 3.** Pathway analysis for the set of genes with SNPs found to be associated with changes on HbF levels in response to HU therapy among the studies included in the systematic review.

| Term | *P*-value | Adjusted *P*-value | Odds  Ratio | Combined Score | Genes |
| --- | --- | --- | --- | --- | --- |
| VEGF ligand-receptor interactions Homo sapiens R-HSA-194313 | 0.00001898 | **0.0002847** | 443.93 | **4826.43** | *FIGF; FLT1* |
| VEGF binds to VEGFR leading to receptor dimerization Homo sapiens  R-HSA-195399 | 0.00001898 | **0.0002847** | 443.93 | **4826.43** | *FIGF; FLT1* |
| Urea cycle Homo sapiens R-HSA-70635 | 0.00003048 | **0.0003048** | 332.92 | **3461.84** | *ARG2; ARG1* |
| Nitric oxide stimulates guanylate cyclase Homo sapiens R-HSA-392154 | 0.02105 | 0.08790 | 51.98 | 200.68 | *ARG2; ARG1* |
| Metabolism of polyamines Homo sapiens R-HSA-351202 | 0.002275 | 0.01707 | 32.36 | 196.93 | *GLP2R; PDE7B* |
| Peroxisomal lipid metabolism Homo sapiens R-HSA-390918 | 0.02271 | 0.08790 | 47.97 | 181.57 | *NOS1* |
| Glucagon-type ligand receptors Homo sapiens R-HSA-420092 | 0.02521 | 0.08790 | 43.00 | 158.28 | *HAO2* |
| OS, RNS production in response to bacteria Homo sapiens R-HSA-1222556 | 0.02852 | 0.08790 | 37.78 | 134.40 | *GLP2R* |
| G alpha (s) signalling events Homo sapiens R-HSA-418555 | 0.006348 | 0.03809 | 18.90 | 95.62 | *NOS1* |
| Ion homeostasis Homo sapiens R-HSA-5578775 | 0.04249 | 0.1062 | 24.92 | 78.70 | *FIGF; FLT1* |
| Class B/2 (Secretin family receptors) Homo sapiens R-HSA-373080 | 0.06908 | 0.1476 | 14.98 | 40.05 | *ARG2; ARG1* |
| Platelet homeostasis Homo sapiens R-HSA-418346 | 0.07146 | 0.1476 | 14.46 | 38.15 | *NOS1* |
| Oxidative Stress Induced Senescence Homo sapiens R-HSA-2559580 | 0.07463 | 0.1476 | 13.81 | 35.85 | *GLP2R* |
| Platelet degranulation Homo sapiens R-HSA-114608 | 0.08563 | 0.1492 | 11.95 | 29.36 | *NOS1* |
| Signaling by VEGF Homo sapiens R-HSA-194138 | 0.03100 | 0.08790 | 8.04 | 27.93 | *MAP3K5* |
| Response to elevated platelet cytosolic Ca2+ Homo sapiens R-HSA-76005 | 0.08953 | 0.1492 | 11.40 | 27.50 | *FIGF; NOS1* |
| Metabolism of amino acids and derivatives Homo sapiens R-HSA-71291 | 0.03223 | 0.08790 | 7.87 | 27.02 | *FIGF* |
| Cardiac conduction Homo sapiens R-HSA-5576891 | 0.1088 | 0.1718 | 9.26 | 20.54 | *FIGF* |
| Cellular Senescence Homo sapiens R-HSA-2559583 | 0.1284 | 0.1926 | 7.74 | 15.89 | *NOS1* |
| Hemostasis Homo sapiens R-HSA-109582 | 0.07871 | 0.1476 | 4.71 | 11.98 | *MAP3K5* |
| Muscle contraction Homo sapiens R-HSA-397014 | 0.1542 | 0.2103 | 6.34 | 11.86 | *FIGF; FLT1; GLP2R; PDE7B* |
| Platelet activation, signaling and aggregation Homo sapiens R-HSA-76002 | 0.1947 | 0.2530 | 4.89 | 8.01 | *NOS1* |
| Cellular responses to stress Homo sapiens R-HSA-2262752 | 0.2702 | 0.3118 | 3.35 | 4.38 | *FIGF* |
| Signal Transduction Homo sapiens R-HSA-162582 | 0.1485 | 0.2103 | 2.19 | 4.18 | *GLP2R; PDE7B* |
| GPCR downstream signaling Homo sapiens R-HSA-388396 | 0.2024 | 0.2530 | 2.58 | 4.13 | *ARG2; ARG1; HAO2* |
| GPCR ligand binding Homo sapiens R-HSA-500792 | 0.3192 | 0.3419 | 2.74 | 3.13 | *MAP3K5* |
| Metabolism Homo sapiens R-HSA-1430728 | 0.2169 | 0.2603 | 2.03 | 3.11 | *GLP2R; PDE7B* |
| Signaling by GPCR Homo sapiens R-HSA-372790 | 0.3017 | 0.3353 | 1.93 | 2.31 | *GLP2R* |
| Metabolism of lipids and lipoproteins Homo sapiens R-HSA-556833 | 0.4344 | 0.4493 | 1.84 | 1.53 | *HAO2* |
| Immune System Homo sapiens R-HSA-168256 | 0.7457 | 0.7457 | 0.75 | 0.22 | *NOS1* |
